# Management system–dependent alterations in colonic mucosal architecture of swine: An Alcian Blue histochemistry and histomorphometric analysis of goblet cell density, epithelial height, and mucin–stromal ratio

**DOI:** 10.14202/vetworld.2026.2379-2392

**Published:** 2026-06-10

**Authors:** Nattawat Chaiyawong, Kwansuda Churud, Peerapong Wanpen, Metaporn Intarachuen, Charkriya Promsuban

**Affiliations:** 1 Siriraj Integrative Center for Neglected Parasitic Diseases, Department of Parasitology, Faculty of Medicine Siriraj Hospital, Mahidol University, Bangkok, Thailand; 2 Professional Laboratory Management Corp Public Company Limited, Bangkok, Thailand; 3 Innotech Laboratory Service Company Limited, Bangkok, Thailand; 4 Bangkok Chain Laboratory and Pathology Clinic, Nonthaburi, Thailand; 5 Department of Anatomy, Faculty of Medical Science, Naresuan University, Phitsanulok, Thailand; 6 Center of Excellence in Medical Biotechnology, Faculty of Medical Science, Naresuan University, Phitsanulok, Thailand

**Keywords:** acidic mucins, Alcian Blue histochemistry, colonic mucosa, goblet cell density, histomorphometry, mucin–stromal ratio, swine management systems, veterinary histology

## Abstract

**Background and Aim:**

Swine management systems can influence intestinal morphology, epithelial organization, and mucosal secretory activity through differences in environmental exposure, nutrition, hygiene, and husbandry practices. However, comparative histological evaluations of colonic mucin architecture among commercial swine production systems remain limited. This study aimed to compare goblet cell density, epithelial height, and mucin–stromal ratio in the colon of swine raised under beta-agonist-free, hygienic, and free-range management systems using Alcian Blue histochemistry and quantitative histomorphometric analysis.

**Materials and Methods:**

Colonic tissue samples were collected from clinically healthy market-weight swine raised under beta-agonist-free, hygienic, and free-range conditions (n = 10 per group). Tissue sections were fixed in 10% neutral buffered formalin, embedded in paraffin, and stained with Alcian Blue (pH 2.5) to visualize acidic mucins. Quantitative histomorphometric analyses were performed using ImageJ/Fiji software to determine goblet cell density, epithelial height, and mucin–stromal ratio. Statistical analysis was performed using one-way analysis of variance followed by Tukey’s honestly significant difference test, and significance was defined as p < 0.05.

**Results:**

Significant differences in colonic mucosal architecture were observed among the three management systems. Swine raised under the beta-agonist-free system exhibited significantly greater epithelial height (252.43 ± 17.27 µm) compared with hygienic (206.27 ± 20.66 µm) and free-range swine (228.84 ± 18.42 µm) (p < 0.05). Goblet cell density was also highest in the beta-agonist-free group (46.76 ± 7.16 cells/field), followed by the free-range and hygienic groups. The mucin–stromal ratio was significantly elevated in beta-agonist-free swine (1.82 ± 0.14) compared with free-range (1.43 ± 0.12) and hygienic swine (1.09 ± 0.10) (p < 0.05). Positive correlations were observed among epithelial height, goblet cell density, and mucin–stromal ratio.

**Conclusion:**

Different swine management systems are associated with measurable alterations in colonic mucosal architecture. Beta-agonist-free swine demonstrated enhanced epithelial development, increased goblet cell abundance, and higher mucin–stromal ratio values, suggesting distinct structural adaptations of the colonic mucosa associated with production practices. The mucin–stromal ratio may serve as a useful integrative histomorphometric indicator for evaluating intestinal mucosal organization in swine.

## INTRODUCTION

Swine production systems vary widely in management intensity, ranging from highly controlled hygienic housing to more extensive free-range environments. These systems differ in housing structure, hygiene management, diet composition, antimicrobial exposure, and environmental microbial load, factors that collectively influence gastrointestinal physiology and mucosal integrity [1]. In Thailand, increasing consumer awareness regarding food safety and animal welfare has contributed to the growing adoption of beta-agonist-free production systems. These systems are often perceived by consumers as more natural or welfare-friendly alternatives to conventional intensive farming, although their biological effects on intestinal structure remain incompletely understood [2].

The large intestine plays a critical role in water and electrolyte absorption and serves as a major interface between the host and a complex microbial ecosystem [3]. Maintenance of colonic epithelial homeostasis depends on coordinated epithelial renewal, mucus secretion, and immune regulation within the mucosal environment [4]. Goblet cells secrete mucins that form a protective mucus layer over the epithelium. Acidic mucins, which can be detected by Alcian Blue staining, contribute to lubrication, epithelial protection, and regulation of host–microbiota interactions [5, 6]. In addition, epithelial height reflects the dynamic balance between epithelial proliferation and shedding and may serve as a morphological indicator of mucosal adaptation to environmental and dietary influences [7]. Disruptions in goblet cell distribution or epithelial architecture may compromise mucosal defense mechanisms and predispose the intestine to inflammation or microbial imbalance [8, 9]. It should also be noted that although histochemical staining allows visualization of intracellular mucins, standard paraffin-based histological processing may partially disrupt the adherent mucus layer on the epithelial surface, which represents a recognized limitation in gastrointestinal histology.

Previous studies have demonstrated that gastrointestinal morphology in swine is sensitive to farming conditions, with variations reported in mucosal thickness, glandular organization, and inflammatory cell distribution across management systems [10, 11]. However, most available investigations have focused primarily on small intestinal morphology, early post-weaning stages, or generalized intestinal histology, whereas detailed comparative analyses of colonic mucin architecture in market-weight swine raised under different commercial production systems remain scarce. Furthermore, previous studies have rarely integrated quantitative histomorphometric parameters associated with both epithelial–secretory activity and stromal organization. In particular, no previous study has quantitatively evaluated the mucin–stromal ratio (MSR) as an integrative indicator reflecting the balance between mucin-producing epithelial compartments and supporting stromal connective tissue within the colonic mucosa. Consequently, important gaps remain in understanding how different husbandry systems influence coordinated epithelial–goblet cell adaptations and mucosal structural organization in the swine colon.

Our recent investigations further demonstrated that husbandry practices can influence histomorphological characteristics in organs beyond the gastrointestinal tract. A previous study reported lobe-specific alterations in pulmonary architecture, fibrosis, and alveolar macrophage distribution among swine raised under different management systems, indicating that production environments may induce systemic tissue-specific adaptations [12]. These findings suggest that organ-specific responses to environmental and management conditions may vary according to tissue function and local physiological demands. Nevertheless, despite growing interest in management-associated histological adaptations, comparative Alcian Blue-based histomorphometric evaluation of the swine colon across beta-agonist-free, hygienic, and free-range systems has not previously been performed. In addition, relationships among epithelial height, goblet cell density, and MSR within the context of different commercial production systems remain poorly characterized.

Therefore, the present study aimed to evaluate colonic mucin characteristics and epithelial architecture using Alcian Blue histochemistry and quantitative histomorphometric analysis in swine raised under beta-agonist-free, hygienic, and free-range management systems. Specifically, the study compared goblet cell density, epithelial height, and MSR among the three production systems to characterize structural adaptations of the colonic mucosa associated with different husbandry practices. This work provides the first quantitative assessment of MSR in the swine colon and offers new insight into coordinated epithelial–goblet cell remodeling and mucosal structural organization in market-weight pigs raised under different commercial management conditions.

## MATERIALS AND METHODS

### Ethical approval

Ethical approval for this study was obtained from the Animal Ethics Committee, Center for Animal Research, Naresuan University, Phitsanulok, Thailand (Approval No. NU-AEE620505). All experimental procedures were conducted in accordance with institutional animal welfare regulations and the Guidelines for the Care and Use of Laboratory Animals of the National Institutes of Health, USA. The study also complied with the ARRIVE 2.0 (Animal Research: Reporting of *In Vivo* Experiments) guidelines to ensure transparent, reproducible, and ethically responsible reporting of animal-related research. Colon samples were collected post-mortem from swine slaughtered for commercial purposes under veterinary supervision, and no animals were euthanized specifically for this investigation. Animals exhibiting gross gastrointestinal lesions, diarrhea, or signs of systemic disease at slaughter were excluded from tissue collection. Tissue handling and sample processing were performed by trained personnel, and all histological and histomorphometric evaluations were conducted by observers blinded to the management system to minimize analytical bias.

### Study period and location

The study was conducted between January 2025 and June 2025 in Thailand. Swine samples were obtained from commercial farms operating under beta-agonist-free, hygienic, and free-range production systems located within the same geographic region to minimize environmental variability. Histological processing, staining procedures, and histomorphometric analyses were performed at the Department of Anatomy, Faculty of Medical Science, Naresuan University, Phitsanulok, Thailand.

### Study design

This study was designed as a comparative cross-sectional histomorphometric investigation evaluating colonic mucosal architecture in market-weight swine raised under three different management systems: beta-agonist-free, hygienic, and free-range conditions. The experimental unit was the individual pig. A total of 30 clinically healthy swine were included, comprising 10 animals per management system. Quantitative histological analyses focused on epithelial height, goblet cell density, and MSR using Alcian Blue histochemistry and digital image analysis. Comparative statistical analyses were subsequently performed to identify management-associated differences in colonic mucosal organization.

### Animal selection and grouping

This comparative histological study was conducted using colonic tissues obtained from clinically healthy, market-weight swine raised under three different management systems in Thailand: beta-agonist-free, hygienic, and free-range production systems. A total of 30 animals (n = 10 per group) were included in the study. Animals were selected from commercial farms representative of each management system within the same geographic region to reduce environmental variability. All animals were maintained continuously within their respective production systems prior to slaughter. The swine were commercial crossbred pigs commonly used in Thai pork production (Thai native × Landrace × Large White). Animals were of comparable production stage at slaughter, with similar market weights and ages across groups, ensuring that histological comparisons were not confounded by developmental differences. To minimize anatomical variability, tissue samples were consistently collected from the spiral colon immediately after slaughter using standardized anatomical landmarks. Collected tissues were promptly fixed for histological processing.

### Housing and management conditions

Swine were raised under three commonly practiced management systems: beta-agonist-free, hygienic, and free-range systems. Housing and management conditions for each system are summarized in Table 1. Briefly, beta-agonist-free swine were maintained in conventional indoor pens without the use of β-agonist growth promoters and followed routine commercial feeding and veterinary management practices. Hygienic swine were raised in controlled indoor facilities with enhanced biosecurity measures, including regulated sanitation procedures and structured veterinary supervision. In contrast, free-range swine were reared under extensive outdoor conditions with continuous access to natural substrates and greater environmental exposure. Although housing design, hygiene practices, and feeding strategies differed among systems, all animals received nutritionally balanced diets appropriate for their growth stage, had ad libitum access to water, and were clinically healthy at the time of slaughter (Table 1).

**Table 1:** Comparative characteristics of the three swine management systems.

Variable	Beta-agonist-free system	Hygienic system	Free-range system
Housing type	Enclosed housing with controlled management and prohibition of beta-agonist feed additives	Intensive indoor housing with enhanced biosecurity measures	Outdoor or semi-outdoor housing with access to open areas
Biosecurity	Standard farm hygiene	Enhanced biosecurity and sanitation protocols	Minimal biosecurity; open environment
Space allowance	~0.8–1.0 m²/pig	~0.8 m²/pig	≥4 m²/pig
Flooring	Concrete or partially slatted floor	Fully slatted or hygienic flooring	Natural soil/grass substrate
Environmental control	Natural ventilation	Controlled ventilation and temperature	Natural environmental exposure
Outdoor access	None	None	Continuous outdoor access
Diet composition	Commercial feed formulated without beta-agonist additives	Standard commercial feed formulation	Commercial feed supplemented with natural forage or environmental feed sources
Feeding regimen	Scheduled feeding with formulated feed	Scheduled feeding with formulated feed	Combination of scheduled feeding and opportunistic foraging
Water supply	Ad libitum	Ad libitum	Ad libitum
Antimicrobial/antiparasitic management	Restricted use, applied only when necessary under veterinary supervision	Preventive and therapeutic use according to farm management protocols	Minimal routine use; treatment applied when clinically indicated
Environmental microbial exposure	Moderate environmental exposure within controlled housing	Low environmental microbial exposure due to strict hygiene and sanitation	Higher environmental microbial exposure from soil and outdoor environment
Animal health status	Clinically healthy at slaughter	Clinically healthy at slaughter	Clinically healthy at slaughter
Weaning age	Approximately 21–28 days	Approximately 21–28 days	Approximately 21–28 days

### Tissue collection and sample preparation

Fresh colon samples were collected immediately post-mortem from swine slaughtered for commercial purposes at local slaughterhouses under veterinary supervision. No animals were sacrificed specifically for this study. Tissue samples were consistently obtained from the mid-portion of the spiral colon, approximately 1–1.5 m distal to the ileocecal junction, using standardized anatomical landmarks to minimize regional variation in colonic histology. Following excision, tissues were gently rinsed with ice-cold phosphate-buffered saline (PBS; pH 7.4) to remove luminal contents while avoiding mechanical disruption of the mucosal surface. Full-thickness tissue segments (approximately 2 × 3 cm) were collected and immediately fixed in 10% neutral buffered formalin (NBF; pH 7.0) for 7 days before histological processing.

### Tissue processing and histological staining

Fixed tissues were processed routinely for paraffin embedding. Briefly, samples were dehydrated through a graded ethanol series, cleared in xylene, and embedded in paraffin wax [13]. Sections of 5 µm thickness were cut using a rotary microtome (Leica RM2235; Leica Biosystems, Wetzlar, Germany) and mounted on poly-L-lysine-coated glass slides (Thermo Fisher Scientific, Waltham, MA, USA). To visualize acidic mucins within goblet cells, sections were stained with Alcian Blue 8GX (Sigma-Aldrich, St. Louis, MO, USA) at pH 2.5 according to established histochemical protocols [14]. Sections were subsequently counterstained with nuclear fast red (Sigma-Aldrich) to provide nuclear contrast and facilitate identification of epithelial structures.

Positive and negative staining controls were included in each staining batch to ensure staining reliability. Porcine duodenal mucosa served as the positive control, whereas negative control sections were processed identically but without the Alcian Blue reagent. To minimize potential batch effects, all slides were stained in a single staining run under identical conditions.

It is recognized that standard formalin fixation and paraffin embedding may result in partial loss or displacement of the adherent mucus layer, particularly in intestinal tissues. Consequently, the mucin measurements obtained in this study primarily reflect intracellular acidic mucins within goblet cells and mucin pools within crypt lumina rather than the intact luminal mucus barrier. Although mucus-preserving fixatives such as Carnoy’s solution or methacarn (Thermo Fisher Scientific) can better preserve the surface mucus layer, these methods were not applied because tissues were collected under commercial slaughterhouse conditions. This limitation was considered during interpretation of mucin-related parameters.

### Histomorphometric analysis

Histological evaluation focused on goblet cell density, epithelial height, and mucin distribution within the colonic mucosa. Quantitative analyses were performed using ImageJ/Fiji software (version 1.54f; National Institutes of Health, Bethesda, MD, USA).

For goblet cell density analysis, Alcian Blue-positive goblet cells were counted within the mucosal epithelium in five randomly selected non-overlapping microscopic fields per section at 20× magnification to ensure clear visualization of stained goblet cells. Results were expressed as the mean number of goblet cells per field.

Epithelial height was measured as the perpendicular distance from the luminal epithelial surface to the basal lamina. Measurements were obtained from 10 well-oriented epithelial regions per field at 10× magnification, allowing visualization of intact epithelial structures. Mean epithelial height values were calculated for each animal.

Quantitative analysis of mucin and stromal components was performed using ImageJ software. High-resolution digital micrographs of Alcian Blue-stained sections were captured under identical imaging conditions, including standardized illumination, magnification, exposure time, and white balance. Images were converted to red-green-blue format and calibrated using the embedded scale bar to ensure accurate area measurements.

To isolate mucin-positive regions, color deconvolution followed by automated thresholding was applied to identify Alcian Blue-positive staining representing acidic mucins within goblet cells and mucus pools. Fixed threshold parameters (hue 180–220; saturation 50–255) were applied uniformly across all images to minimize analytical variability. Regions of interest were manually delineated using the freehand selection tool to encompass the lamina propria of the mucosa while excluding epithelial cells, glandular lumina, and muscular layers to ensure accurate identification of stromal connective tissue.

The mucin-positive area and stromal connective tissue area were quantified using the Analyze → Measure function. Analyses were conducted on five non-overlapping fields per section and five sections per animal (25 fields per animal), and mean values were used for statistical analysis.

To further characterize mucosal architecture, we developed and applied the MSR, calculated as the ratio of Alcian Blue-positive mucin area to stromal connective tissue area within the lamina propria. This integrative index provides a quantitative measure of the balance between mucin secretion and stromal structural support in the colonic mucosa.

All histomorphometric measurements were performed by two independent observers blinded to the management system. Measurement reproducibility was assessed using the coefficient of variation, which was below 5% for all evaluated parameters, indicating high analytical consistency. Standardized image acquisition and analysis parameters were applied to all samples to ensure reproducibility across the dataset.

### Statistical analysis

All quantitative data are expressed as mean ± standard error of the mean. Before statistical testing, data normality was assessed using the Shapiro–Wilk test, and homogeneity of variance was evaluated using Levene’s test. Differences among the three management systems were analyzed using one-way analysis of variance. When significant differences were detected, Tukey’s honestly significant difference test was applied for pairwise comparisons between groups.

Effect sizes were calculated using partial eta-squared to estimate the magnitude of group differences. A priori sample size estimation was conducted using G*Power software (version 3.1; Heinrich Heine University Düsseldorf, Düsseldorf, Germany; G*Power Official Website; https://www.psychologie.hhu.de/arbeitsgruppen/allgemeine-psychologie-und-arbeitspsychologie/gpower) assuming an effect size of f = 0.5, significance level α = 0.05, and statistical power of 0.80, indicating that 10 animals per group would provide sufficient statistical power to detect biologically meaningful differences in histomorphometric parameters.

Pearson’s correlation analysis was performed to examine relationships among epithelial height, goblet cell density, and MSR. Correlation analyses were conducted on pooled datasets across all animals, with additional group-stratified exploratory analyses performed to evaluate whether similar relationships were observed within each management system. Statistical significance was defined as p < 0.05, and exact p-values are reported where applicable.

All statistical analyses were performed using SPSS Statistics software (version 27.0; IBM Corp., Armonk, NY, USA) and GraphPad Prism software (version 10.0; GraphPad Software, San Diego, CA, USA). Raw data are available as Supplementary Data.

## RESULTS

### Gross morphology of the large intestine among different swine management systems

**Figure 1:**
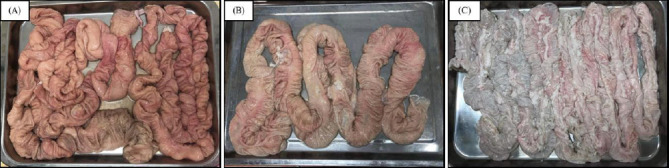
Gross morphology of the large intestine in swine reared under different management systems. Representative colonic images from swine raised under (A) beta-agonist-free, (B) hygienic, and (C) free-range management systems. Visual differences in overall wall appearance and folding patterns can be observed among the groups. The beta-agonist-free samples generally show a thicker and more compact appearance with more distinct segmental folds, whereas the hygienic and free-range samples appear comparatively smoother with less prominent folding. These images are presented for qualitative comparison only and were not subjected to quantitative morphometric analysis. Images are representative of samples from each group (n = 10 per group).

Gross examination of colonic samples revealed visible differences in external morphology among beta-agonist-free, hygienic, and free-range swine (Figure 1). These observations were qualitative because no direct morphometric measurements of intestinal wall thickness were obtained during sampling. In the beta-agonist-free group, the colon exhibited a relatively thick and compact appearance characterized by pronounced segmental folding and a firm intestinal wall. Surface coloration was generally uniform, and the colonic structure demonstrated well-defined convolutions (Figure 1A).

In comparison, colons from hygienic swine demonstrated a moderately thick intestinal wall with smoother and more regular folds than those observed in the beta-agonist-free group (Figure 1B). The tissue appeared less compact, with a more uniform tubular configuration and reduced surface irregularity. In contrast, the free-range swine colon displayed a thinner and more elongated morphology accompanied by wider luminal distension and looser folding patterns. The intestinal wall appeared softer and less compact, and greater variability in surface morphology was observed along the length of the colon (Figure 1C).

Overall, although the general anatomical organization of the colon was preserved across all management systems, visible differences in wall thickness, folding pattern, and tissue compactness were evident. These observations extend our previous investigations of the stomach and lung, suggesting that different swine management systems may induce organ-specific structural adaptations throughout the gastrointestinal tract.

### Histological organization of the large intestine

**Figure 2:**
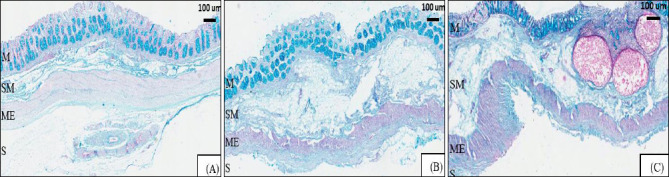
Histological organization of the large intestine in swine reared under different management systems. Representative photomicrographs of Alcian Blue-stained colonic sections from swine raised under (A) beta-agonist-free, (B) hygienic, and (C) free-range management systems. The mucosa (M), submucosa (SM), muscularis externa (ME), and serosa (S) are identifiable in all groups, indicating preservation of overall colonic architecture. Alcian Blue-positive goblet cells (blue staining) illustrate mucin distribution within the epithelial lining. Images are representative of samples from each group (n = 10 per group).

Histological examination of the large intestine from beta-agonist-free, hygienic, and free-range swine at low magnification (2.5×) demonstrated a typical colonic wall organization consisting of four distinct layers in all groups (Figure 2). The mucosa was composed of a simple columnar epithelium containing numerous goblet cells supported by a lamina propria consisting of connective tissue, lymphoid nodules, and a thin muscularis mucosae. The submucosa consisted of dense irregular connective tissue containing numerous blood vessels and interspersed adipose tissue, whereas the muscularis externa comprised inner circular and outer longitudinal smooth muscle layers. The outermost serosa was formed by a thin connective tissue layer.

**Figure 3:**
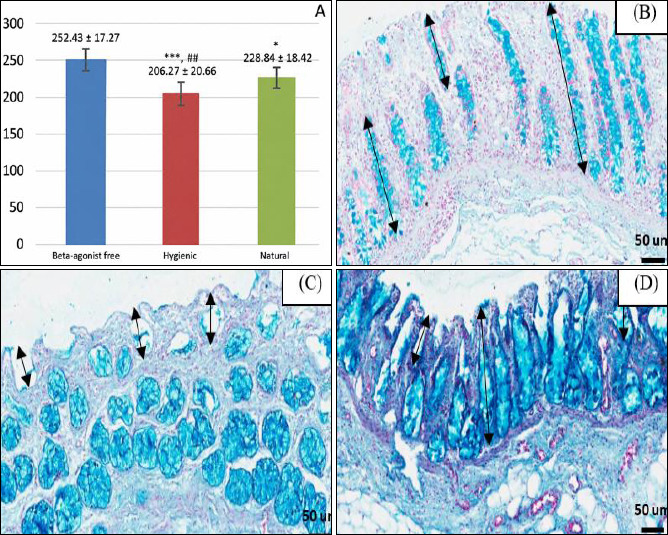
Epithelial height variations among different swine management systems. (A) Quantitative analysis of colonic epithelial height demonstrating significant differences among beta-agonist-free, hygienic, and free-range swine. Epithelial height was significantly greater in the beta-agonist-free group than in the hygienic and free-range groups (***p < 0.0001 and *p < 0.05, respectively) and was also greater in the free-range group than in the hygienic group (##p < 0.01). Data are presented as mean ± standard error or the mean (n = 10 per group). Representative Alcian Blue-stained sections of colonic mucosa from (B) beta-agonist-free, (C) hygienic, and (D) free-range swine highlighting the epithelial layer. Double-headed arrows indicate epithelial height measured from the luminal surface to the basement membrane.

**Figure 4:**
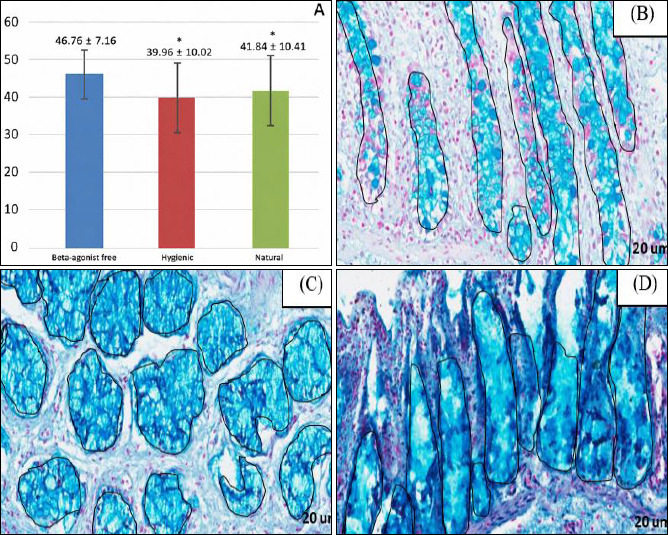
Goblet cell density and mucin distribution in the large intestine among different swine management systems. (A) Quantitative analysis of goblet cell density in the colonic mucosa demonstrating significant differences among beta-agonist-free, hygienic, and free-range swine. Goblet cell density was highest in the beta-agonist-free group compared with the hygienic and free-range groups (*p < 0.05). Data are presented as mean ± standard error of the mean (n = 10 per group). Representative Alcian Blue-stained colonic mucosa from (B) beta-agonist-free, (C) hygienic, and (D) free-range swine highlighting mucin-producing goblet cells and mucin distribution within the epithelium. Black outlined circles indicate areas of mucin accumulation within the mucosal layer.

Although these four layers were consistently observed across all groups, differences in mucosal characteristics were evident among the three management systems. Quantitative analysis demonstrated significant variation in mucosal epithelial architecture. Measurements obtained at 10× magnification revealed significant differences in epithelial height among the management systems (Figure 3). The beta-agonist-free group exhibited the greatest epithelial height, followed by the free-range group, whereas the hygienic group showed the lowest values (p < 0.05–0.001). At higher magnification (20×), goblet cell density within the mucosal epithelium also differed significantly among groups (Figure 4). The highest goblet cell density was observed in beta-agonist-free swine, followed by free-range swine, whereas the hygienic group exhibited the lowest density.

### Epithelial height variations among different management systems

Quantitative analysis of epithelial height within the colonic mucosa revealed significant differences among the three swine management systems (Figure 3). The beta-agonist-free group exhibited the greatest epithelial height (252.43 ± 17.27 µm), which was significantly higher than that observed in the hygienic group (206.27 ± 20.66 µm; p < 0.001) and the free-range group (228.84 ± 18.42 µm; p = 0.03). In addition, epithelial height in the free-range group was significantly greater than that in the hygienic group (p = 0.01).

Overall, epithelial height differed significantly across the management systems, with the highest values observed in beta-agonist-free swine, intermediate values in free-range swine, and the lowest values in hygienic swine.

### Goblet cell density and mucin distribution

Quantitative evaluation of goblet cell density within the colonic mucosa revealed significant differences among the three swine management systems (Figure 4). The beta-agonist-free group exhibited the highest goblet cell density (46.76 ± 7.16 cells/field), which was significantly greater than that observed in the hygienic group (32.96 ± 10.02 cells/field; p = 0.05) and the free-range group (41.84 ± 10.41 cells/field; p = 0.05). Although the free-range group demonstrated a higher mean goblet cell density than the hygienic group, this difference was not statistically significant.

**Figure 5:**
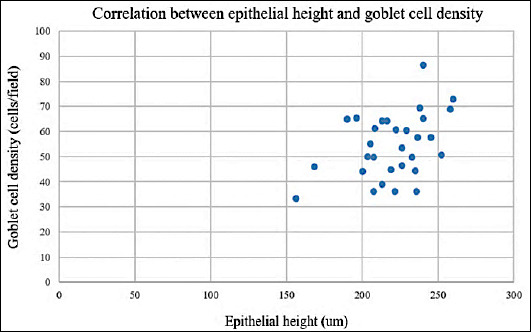
Correlation between epithelial height and goblet cell density in the colonic mucosa of swine raised under different management systems. Scatter plot illustrating the relationship between epithelial height (µm) and goblet cell density (cells/field) measured in colonic mucosal samples from swine raised under beta-agonist-free, hygienic, and free-range management systems (n = 30 total samples; 10 per group). Each point represents an individual sample. A strong positive correlation was observed between epithelial height and goblet cell density (Pearson’s r = 0.82, p < 0.001), indicating that samples with greater epithelial height tended to exhibit higher goblet cell densities.

Pearson’s correlation analysis revealed a strong positive relationship between epithelial height and goblet cell density (r = 0.82, p < 0.001) when pooled individual measurements from all samples were analyzed (n = 30) (Figure 5). Samples with greater epithelial height tended to exhibit higher densities of Alcian Blue-positive goblet cells. Notably, samples from the beta-agonist-free group clustered toward the upper range of both parameters, followed by the free-range group and subsequently the hygienic group. This pattern suggests coordinated mucosal remodeling in which increased epithelial height is associated with enhanced goblet cell abundance and mucin-secretory capacity.

### Mucin–stromal ratio analysis

**Figure 6:**
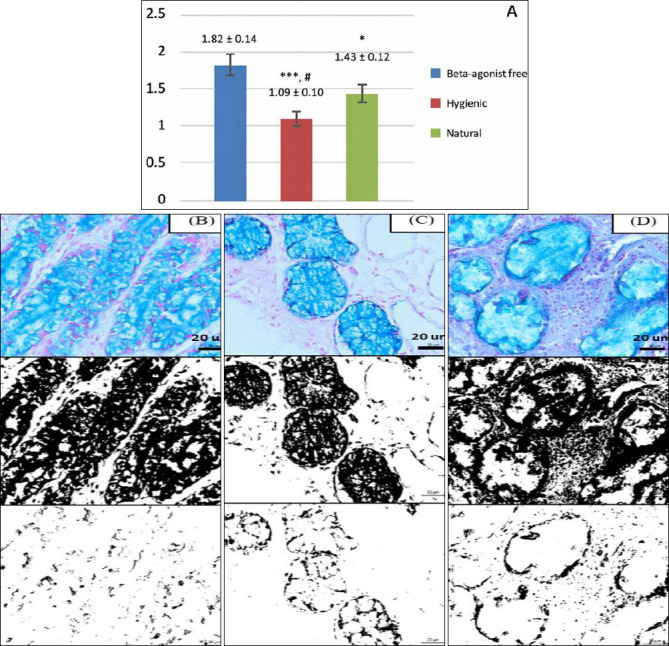
MSR analysis of the colonic mucosa in swine under different management systems. (A) Quantitative analysis of MSR among beta-agonist-free, hygienic, and free-range swine. The MSR was significantly greater in the beta-agonist-free group than in the hygienic and free-range groups (***p < 0.0001 and *p < 0.05, respectively) and was also greater in the free-range group than in the hygienic group (#p < 0.05). Data are presented as mean ± standard error of the mean (n = 10 per group). (B–D) Representative Alcian Blue-stained colonic sections from (B) beta-agonist-free, (C) hygienic, and (D) free-range swine are shown. For each management system, three panels are presented. The upper panel displays the original histological image stained with Alcian Blue (pH 2.5), highlighting acidic mucins within goblet cells and mucus pools. The middle panel shows the ImageJ-processed image following color deconvolution and thresholding used to identify Alcian Blue-positive mucin areas. The lower panel illustrates delineation of stromal connective tissue within the lamina propria used for MSR calculation after exclusion of epithelial structures and glandular lumina. These processed images demonstrate the analytical workflow used to quantify mucin-positive and stromal areas for calculation of MSR.

Quantitative image analysis of Alcian Blue-stained colonic sections revealed significant differences in MSR among the three swine management systems (Figure 6). In this study, MSR is introduced as a novel histomorphometric parameter integrating mucin-secretory activity with stromal structural organization within the colonic mucosa.

The beta-agonist-free group exhibited the highest MSR (1.82 ± 0.14), which was significantly greater than that observed in both the free-range group (1.43 ± 0.12; p < 0.05) and the hygienic group (1.09 ± 0.10; p < 0.05). The free-range group demonstrated an intermediate MSR value that was significantly greater than that of the hygienic group (p < 0.05).

Histologically, higher MSR values corresponded to a greater proportion of Alcian Blue-positive mucin within intestinal glands and along the epithelial surface, whereas lower MSR values reflected a relatively greater proportion of stromal connective tissue within the lamina propria. Correlation analysis further demonstrated that MSR values were positively associated with both goblet cell density and epithelial height across samples. Measurements from the beta-agonist-free group predominated at the higher ranges of these parameters, followed by the free-range and hygienic groups.

Together, these findings suggest that differences in swine management systems may influence the balance between mucin secretion and stromal organization within the colonic mucosa, providing an integrative structural indicator of mucosal functional adaptation.

## DISCUSSION

### Management system-associated changes in colonic mucosal architecture

This study provides new histological evidence that swine management systems differentially influence the architecture of the colonic mucosa in market-weight pigs. Although the fundamental structural organization of the colonic wall, comprising mucosa, submucosa, muscularis externa, and serosa, was preserved across beta-agonist-free, hygienic, and free-range animals, significant quantitative differences were observed in epithelial height, goblet cell density, and MSR. Among these parameters, MSR represents a newly proposed integrative histomorphometric indicator that captures the relative balance between mucin-secreting epithelial components and stromal connective tissue within the lamina propria. By integrating secretory and structural features of the mucosa, this parameter provides complementary information beyond traditional measurements such as epithelial height or goblet cell counts alone [15].

### Gross morphological differences among management systems

Gross anatomical observations suggested visible differences in colonic morphology among the three management systems. Beta-agonist-free swine exhibited a more compact and folded colonic appearance, whereas free-range animals showed a thinner and more elongated morphology, and hygienic swine displayed intermediate characteristics [16]. Because these observations were qualitative and not supported by direct morphometric measurements of wall thickness, they should be interpreted cautiously [17]. Nevertheless, macroscopic differences in intestinal appearance may reflect underlying variations in luminal content, tissue hydration, or mucosal activity, which are known to influence intestinal morphology under different environmental and nutritional conditions [10, 11].

### Epithelial height and mucosal remodeling

At the microscopic level, epithelial height differed significantly among management systems, with the greatest values observed in beta-agonist-free swine, intermediate values in free-range animals, and the lowest values in hygienic swine [18]. Increased epithelial height is commonly associated with expansion of the mucosal surface and changes in epithelial turnover dynamics [19]. However, because functional parameters such as nutrient absorption rates, epithelial proliferation markers, or barrier permeability were not measured in this study, these structural differences should not be interpreted as direct indicators of improved intestinal performance or health [20].

### Goblet cell density and mucin-related structural adaptation

Goblet cell density showed a similar distribution pattern across the management systems. Beta-agonist-free swine exhibited the highest goblet cell counts, followed by free-range animals, whereas hygienic swine displayed the lowest values. Goblet cells are responsible for secreting acidic mucins that form the protective mucus layer covering the intestinal epithelium [21]. Although increased goblet cell density may indicate greater mucus production capacity, the present study did not directly measure mucus secretion rates, mucin gene expression, or barrier integrity [22]. Therefore, the observed differences represent structural variations rather than confirmed functional changes in mucosal defense [23, 24].

The strong positive correlation observed between epithelial height and goblet cell density suggests coordinated epithelial remodeling within the colonic mucosa [4]. Samples with taller epithelium tended to exhibit greater goblet cell abundance, indicating that epithelial expansion and secretory differentiation may occur concurrently [25]. Such coordinated responses are consistent with current understanding of intestinal epithelial biology, in which stem cell activity and differentiation into absorptive and secretory lineages are regulated by shared signaling pathways influenced by luminal stimuli and microbial interactions [26].

### MSR as an integrative histomorphometric indicator

MSR further illustrated how swine management systems influence the structural balance of the colonic mucosa. Beta-agonist-free swine exhibited the highest MSR values, indicating a relatively larger mucin-rich epithelial compartment compared with stromal connective tissue [27]. Free-range animals showed intermediate values, whereas hygienic swine demonstrated the lowest ratios. Because MSR integrates both mucin distribution and stromal architecture, it provides a useful structural indicator of epithelial–stromal organization within the mucosa [25, 28–30]. However, it should be emphasized that this parameter reflects histological composition rather than direct functional performance of the mucus barrier [31].

### Organ-specific responses to husbandry conditions

Interestingly, these colonic findings contrast with observations from our recent investigation of pulmonary tissues in swine raised under similar management conditions, where beta-agonist-free animals showed increased fibrotic remodeling in lung tissue. The coexistence of different structural responses across organs suggests that husbandry practices may exert organ-specific effects, reflecting the complex physiological interactions between environmental exposure, diet, metabolism, and tissue-specific adaptive responses [32].

The relatively lower epithelial height, goblet cell density, and MSR observed in hygienic swine may reflect differences in environmental stimulation, dietary composition, or microbial exposure. Highly controlled housing conditions typically reduce contact with environmental microorganisms and pathogens, which may alter mucosal signaling pathways involved in epithelial turnover and secretory differentiation [33, 34]. However, because microbiome composition, pathogen load, and parasitological status were not examined in this study, this interpretation remains hypothetical and should be investigated in future work.

### Integration with previous gastrointestinal and pulmonary findings

The present findings also complement our previous studies examining gastrointestinal morphology under different swine management systems. Earlier investigations of gastric and intestinal tissues suggested that environmental and nutritional conditions can influence mucosal structure along different regions of the digestive tract [10, 11]. To reconcile these observations with our previous studies on gastric and pulmonary histology, it should be noted that morphological responses to management systems appear to be organ-specific.

While the colon of beta-agonist-free swine exhibited a relatively thicker and more compact gross appearance in the present study, our earlier investigations of the stomach and lung demonstrated variations in specific tissue layers rather than uniform thickening across the entire organ. These differences likely reflect the distinct physiological roles and structural organization of each organ system. The colon, as a site of mucosal secretion, microbial interaction, and luminal transport, may respond to management conditions primarily through changes in epithelial development and mucin production, whereas gastric and pulmonary tissues exhibit adaptations related to their respective digestive and respiratory functions. Collectively, these findings suggest that swine management systems influence tissue morphology across multiple organs, although the specific structural manifestations vary according to organ function and local environmental exposure.

### Limitations and future directions

Several limitations should be considered when interpreting the results of this study. First, histological processing may lead to partial loss or redistribution of mucus, potentially affecting quantitative measurements of mucin-rich areas. Second, the study focused exclusively on structural histology and did not include functional assessments such as intestinal permeability, immune signaling, microbiome composition, or production performance parameters. Third, biological variables such as sex, genetic background, and detailed dietary composition were not controlled across farms and may have influenced mucosal characteristics. Fourth, the study design involved single time-point sampling at market-weight, which prevents evaluation of temporal changes in mucosal adaptation during development or growth. Finally, gross anatomical observations were qualitative and were not supported by direct morphometric measurements of intestinal wall thickness.

Future investigations integrating histology with microbiome profiling, parasitological screening, immunological markers, and functional barrier assays would provide a more comprehensive understanding of how different swine management systems influence intestinal health and physiology.

## CONCLUSION

This study demonstrated that different swine management systems are associated with significant variations in the histomorphological organization of the colonic mucosa in market-weight pigs. Quantitative histomorphometric analysis revealed that beta-agonist-free swine exhibited significantly greater epithelial height, higher goblet cell density, and elevated MSR values compared with hygienic and free-range systems. Free-range swine generally demonstrated intermediate values, whereas hygienic swine consistently exhibited the lowest measurements for most evaluated parameters. In addition, positive correlations among epithelial height, goblet cell density, and MSR indicated coordinated epithelial–secretory remodeling within the colonic mucosa.

The findings suggest that production environments may influence the structural balance between mucin-secreting epithelial compartments and stromal connective tissue within the colon. From a practical perspective, these histomorphological differences may contribute to variation in mucosal organization, epithelial adaptation, and mucus-associated barrier structure among commercial swine production systems. The introduction of MSR as a novel quantitative histomorphometric parameter provides an additional integrative approach for evaluating mucosal architecture and may serve as a useful supplementary indicator in future gastrointestinal histology studies involving livestock species.

A major strength of this study was the integration of conventional histology with standardized digital histomorphometric analysis to evaluate multiple complementary parameters of mucosal organization. The use of blinded quantitative assessment, standardized image acquisition protocols, and comparative evaluation across three commercially relevant management systems further enhanced analytical consistency and biological relevance. In addition, this investigation provides the first quantitative application of MSR in the swine colon and extends previous observations of management-associated tissue adaptations beyond the stomach and pulmonary system.

Nevertheless, the study was limited by the absence of functional assessments such as microbiome profiling, intestinal permeability testing, mucin gene expression analysis, and immunological evaluation. Furthermore, qualitative gross anatomical observations were not supported by direct morphometric measurements of intestinal wall thickness. Future studies integrating histology with molecular, microbiological, and functional analyses are warranted to clarify the biological mechanisms underlying these structural adaptations and their potential implications for intestinal physiology and swine health.

In conclusion, swine management systems are associated with measurable differences in colonic mucosal architecture, particularly in epithelial height, goblet cell density, and MSR. Beta-agonist-free production systems demonstrated the most prominent mucosal structural development among the evaluated groups. These findings improve current understanding of management-associated intestinal adaptations in swine and highlight the value of integrative histomorphometric approaches for investigating gastrointestinal tissue organization under different production conditions.

## DATA AVAILABILITY

All data generated or analyzed during this study are included in this published article. Raw histological images and quantitative datasets used to support the findings of this study are available from the corresponding author upon reasonable request.

## AUTHORS’ CONTRIBUTIONS

NC: Drafted and revised the manuscript. KC, PW and MI: Conducted laboratory experiments, collected, analyzed and interpreted data. CP: Conceived and designed the study, interpreted data, and critically drafted and revised the manuscript. All authors have read and approved the final version of the manuscript.
